# Neuroendocrine signaling pathways and the nutritional control of puberty in heifers

**DOI:** 10.21451/1984-3143-AR2018-0013

**Published:** 2018-08-03

**Authors:** Rodolfo C. Cardoso, Bruna R.C. Alves, Gary L. Williams

**Affiliations:** 1 Texas A&M University, College Station, TX, USA; 2 University of Nevada, Reno, NV,USA; 3 Texas A&M AgriLife Research, Beeville, TX, USA

**Keywords:** heifers, hypothalamus, leptin, nutrition, puberty.

## Abstract

Puberty is a complex physiological process in females that requires maturation of the reproductive neuroendocrine system and subsequent initiation of high- frequency, episodic release of gonadotropin-releasing hormone (GnRH) and luteinizing hormone (LH). Genetics and nutrition are two major factors controlling the timing of puberty in heifers. While nutrient restriction during the juvenile period delays puberty, accelerated rates of body weight gain during this period have been shown to facilitate pubertal development by programming hypothalamic centers that underlie the pubertal process. Among the different metabolic factors, leptin plays a critical role in conveying nutritional information to the neuroendocrine axis and controlling pubertal progression. Because GnRH neurons are devoid of the leptin receptor, leptin’s effects on GnRH neurons must be relayed via an afferent neuronal network. Two neuronal populations located in the arcuate nucleus (ARC) that express the orexigenic peptide neuropeptide Y (NPY), and the anorexigenic peptide alpha melanocyte-stimulating hormone (αMSH), are key components of afferent pathways that convey inhibitory (NPY) and excitatory (αMSH) inputs to GnRH neurons. In addition, ARC neurons expressing kisspeptin, a potent stimulator of GnRH release, are also involved in the nutritional regulation of puberty. Our studies have demonstrated that increased planes of nutrition during juvenile development result in morphological and functional changes in hypothalamic pathways comprising NPY, proopiomelanocortin (POMC), and kisspeptin neurons. Changes included differential expression of NPY, POMC, and Kiss1 in the ARC, and plasticity in the axonal projections to GnRH and kisspeptin neurons. Additionally, increased rates of body weight gain also promoted changes in the pattern of DNA methylation, a key epigenetic mechanism for regulation of gene expression. Finally, our most recent findings suggest that maternal nutrition during gestation can also induce structural and functional changes in hypothalamic neurocircuitries that are likely to persist long after pubertal maturation and influence reproductive performance throughout adulthood in cattle.

## Introduction

Pubertal maturation in female mammals is an intricate physiological process that involves physical and behavioral changes associated with activation of the hypothalamic-pituitary-ovarian axis and subsequent establishment of reproductive cyclicity (Sisk and Foster, 2004). Reproductive maturation is initiated primarily at the hypothalamic level by the acceleration of gonadotropin-releasing hormone (GnRH) secretion from GnRH neurons. The increase in pulsatile release of GnRH and subsequent rise in luteinizing hormone (LH) pulse frequency support the final maturation of ovarian follicles and steroidogenesis that are required for first ovulation (Ryan and Foster, 1980). The process of pubertal development is largely controlled by genetic and environmental factors, among which nutrition plays a prominent role. Data in humans and animals unequivocally demonstrate that increased nutrient intake during peripubertal development facilitates reproductive maturation in females (Ryan and Foster, 1980; Amstalden *et al*., 2011).

The timing of pubertal development has important implications for livestock production. In beef heifers, lifetime productivity is heavily dependent upon their ability to reach reproductive competence, to conceive early during their first breeding season, and to calve the first time by approximately 24 months of age (Lesmeister *et al*., 1973). Moreover, the incidence of multiple estrous cycles before the first breeding positively influences yearling fertility (Short and Bellows, 1971). However, a significant proportion of beef heifers within existing U.S. production systems fail to reach the developmental end-points necessary to achieve these objectives (Hughes, 2013). This is particularly true for later-maturing breeds (*e.g., Bos indicus*-influenced) in which the skeletal size required to support a healthy and safe pregnancy is frequently attained well before the establishment of regular estrous cycles. Therefore, a better understanding of the neuroendocrine mechanisms controlling puberty can assist in the development of novel managerial strategies that exploit brain plasticity during critical windows of development and lead to strategies for successfully programming the onset of puberty around 12 to 14 months of age.

The objectives of this review are to present an overview of the neuroendocrine processes controlling puberty in heifers, discuss the effects of nutrition on these processes, and summarize recent research findings regarding the programming effects of nutrition during early development on hypothalamic pathways that control reproduction. While this review focuses primarily on pubertal development in heifers, supportive data from other species including sheep and rodents are also discussed.

## Neuroendocrine control of reproductive maturation

### Estradiol negative feedback on gonadotropin secretion

The onset of puberty in the heifer is characterized by a marked increase in the frequency of LH pulses that first becomes apparent at approximately 50 days before first ovulation. This characteristic increase serves as the most accurate predictor of pubertal onset (Day *et al*., 1984) and is resultant of coincident increases in pulsatile secretion of GnRH (Cardoso *et al*., 2014a). As frequency of LH pulses increases, their amplitude declines. However, despite the absence of consistent changes in pulse frequency or amplitude prior to 50 days, a trend for mean circulating concentrations of LH to increase has been detected in the heifer as early as 4 months preceding puberty (Swanson *et al*., 1972; Schams *et al*., 1981; Day *et al*., 1984). The heightened frequency of LH pulses reflects maturational changes of hypothalamic centers responsible for the pulsatile mode of GnRH release (Sizonenko and Aubert, 1986). These changes are highlighted by a marked decline in negative feedback responsiveness to estradiol-17β and are similar to those observed in the ewe lamb (Foster and Ryan, 1979). Although the synthesis and secretion of gonadotropins appear to be largely gonadal steroid-independent during the early postnatal period, a gonad-dependent suppression of GnRH/LH develops during the juvenile period and reflects an increase in responsiveness to estradiol negative feedback (Day *et al*., 1984, 1986; Foster and Ryan, 1979). Ovariectomy at this time, without estradiol replacement, results in establishment of high-frequency pulses of GnRH/LH, typical of those at puberty. Conversely, estradiol replacement prevents the castration-induced rise in GnRH/LH by restoring the inhibitory tone characteristic of the prepubertal state. Estradiol-mediated inhibition persists until hypothalamic changes associated with maturation of the reproductive neuroendocrine axis occur, at which time the GnRH pulse generator escapes from estradiol negative feedback in sheep (Foster and Ryan, 1979). Importantly, GnRH neurons do not contain estrogen receptor-alpha (ESR1; Lehman *et al*., 1993). Therefore, modifications in responsiveness to estradiol negative feedback are not mediated directly by estradiol at the level of the GnRH neuron. Moreover, although both follicle-stimulating hormone (FSH) and LH are ultimately controlled by GnRH from the hypothalamus, the change in negative feedback responsiveness to estradiol does not appear to result in a measurable modification in secretion patterns of FSH preceding puberty in heifers (Schams *et al*., 1981) or ewe lambs (Foster *et al*., 1975). Thus, a limitation in availability of FSH is not a primary factor regulating the timing of puberty in ruminants. Once pulses of LH occur at an interval of every 40 to 50 min, circulating concentrations of LH increase markedly and result in heightened stimulation of ovarian follicles, an increase in circulating concentrations of estradiol, and initiation of a LH surge that induces first ovulation or luteinization of a large follicle (Kinder *et al*., 1987). The estradiol-induced surge of LH occurs through a parallel positive feedback effect of estradiol at hypothalamic components that mediate a prolonged surge of GnRH. However, the ability of estradiol to induce a surge release of LH becomes functional between 3 and 5 months of age in heifers (Staigmiller *et al*., 1979) and thus is operable well before puberty.

### Neuronal processes underlying the change in negative feedback responsiveness to estradiol

The neuronal network in the hypothalamus has the inherent ability to produce a pulsatile pattern of GnRH release and depends upon synchronous firing of GnRH neurons (Funabashi *et al*., 2000). However, since GnRH neurons do not contain ESR1, the role of changing responsiveness to estradiol negative feedback in modulating the GnRH pulse generator as puberty approaches has not been clearly delineated. Nonetheless, studies in mice have clearly shown that ESR1 is the major estrogen receptor mediating estradiol negative feedback effects on GnRH secretion (Dorling *et al*., 2003). Moreover, based on work in rodents, neurons located in the arcuate nucleus (ARC) that contain ESR1 are essential for communicating estradiol negative feedback (Bronson, 1981). In this context, it has been proposed that kisspeptin neurons are responsible for mediating the synchronized firing of GnRH neurons (Navarro *et al*., 2009; Qiu *et al*., 2016), underlie the estradiol feedback regulation of GnRH secretion (Dubois *et al*., 2016), and thus greatly influence pubertal maturation (Mayer *et al*., 2010; Redmond *et al*., 2011a, b) in both rodents and ruminants. Kisspeptin, a member of the RF-amide related peptide (RFRP) superfamily, is controlled by the *Kiss1* gene and its receptor (KISS1R), and is expressed in a variety of tissues, including the hypothalamus. Moreover, KISS1R colocalizes with GnRH neurons and is responsible for regulating the release of GnRH (Mayer *et al*., 2010). Gene mutations resulting in loss of this signaling pathway result in the failure to attain puberty in primates (Terasawa *et al*., 2013). Additional variants of Kiss1-containing cells have been localized specifically within the ARC and are termed KNDy neurons. Kisspeptin cells localized to the ARC co- express two other neuronal peptides, neurokinin B (NKB) and dynorphin. KNDy neurons express receptors for both NKB and dynorphin, but do not contain kisspeptin receptors. Thus, KNDy cells function as a signaling network, secreting kisspeptin in response to their own release of NKB which stimulates release of GnRH by its direct action on both cell bodies and nerve terminals of GnRH neurons (Navarro *et al*., 2009; Lehman *et al*., 2010). Dynorphin is then released which terminates KNDy neuron activity. It has been proposed that the repeating nature of this paradigm provides, for the first time, a plausible explanation of cellular activity that represents the physical source of the GnRH pulse generator (Lehman *et al*., 2010; Goodman *et al*., 2013). Deletion of ESR1 in all kisspeptin-expressing neurons in a rodent model advances the onset of puberty, supporting the idea that ESR1 has the inherent ability to suppress GnRH/LH secretion and pubertal onset by its actions on kisspeptin neurons (Cheong *et al*., 2015). The interrelationship between ESR1 and kisspeptin neurons provides a potential basis through which changes in negative feedback responsiveness to estradiol could regulate the timing of puberty in heifers and ewe lambs. However, the exact mechanism remains elusive. Day *et al*. (1987) observed a reduction in the overall number of estradiol receptors in the mediobasal hypothalamus in intact heifers approaching puberty, similar to findings reported during juvenile development in rats (Kato *et al*., 1974). In contrast, Bedenbaugh *et al*. (2018) reported that the peripubertal increase in LH pulsatility in ovariectomized, estradiol-treated ewe lambs was associated with enhanced ESR1 mRNA expression in kisspeptin neurons in the ARC. Moreover, the absence of estradiol in ovariectomized ewe lambs was associated with the greatest ESR1 mRNA abundance and percentage of kisspeptin neurons containing ESR1 protein in the ARC. Therefore, changes in the expression of ESR1, particularly in kisspeptin neurons in the ARC, fail to explain the pubertal escape from estradiol negative feedback in the ruminant model.

## Impact of nutrition on pubertal development

### Nutritional acceleration of puberty

The important effects of nutrition controlling the reproductive neuroendocrine system and pubertal progression in heifers have been well established. Previous experiments conducted by our group (Cardoso *et al*., 2014a, b; Allen *et al*., 2017) and others (Gasser *et al*., 2006a) have demonstrated that increasing nutrient intake during the juvenile period can markedly advance the timing of puberty in beef heifers. In studies performed by Gasser *et al*. (2006a), the majority of heifers weaned at approximately 3.5 months of age and fed high-concentrate diets to achieve accelerated rates of body weight gain exhibited puberty before 300 days of age (precocious puberty). Although those studies were performed in *Bos taurus* breeds (Angus and Hereford), similar findings were observed in our studies using *Bos indicus*-influenced heifers, which are later maturing. In these studies, heifers (1/2 Angus × 1/4 Hereford × 1/4 Brahman) were weaned between 3.5 and 4 months of age and fed a high-concentrate diet to promote a rate of body weight gain of approximately 1 kg/day (Cardoso *et al*., 2014a, b). This dietary regimen was shown to significantly advance puberty, with a high percentage (~85%) of heifers reaching puberty before 12 months of age.

**Figure 1:**
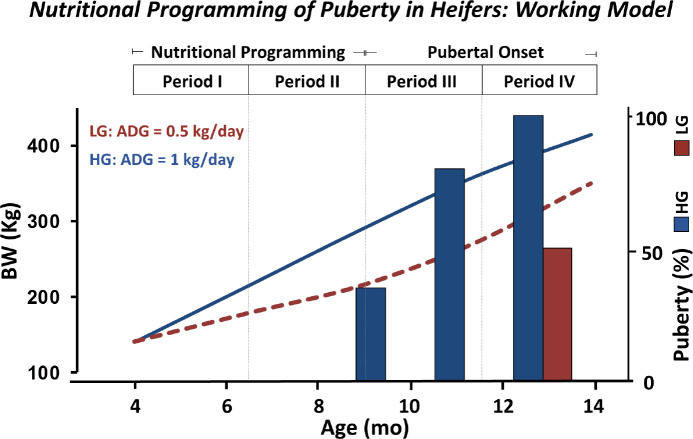
Schematic diagram of the working model for the nutritional programming of puberty in heifers. Heifers weaned at approximately 3.5 months of age and fed a high-concentrate diet to promote a relative high rate of body weight gain (1 kg/day; blue line) attain puberty significantly earlier (blue bars) when compared to heifers gaining body weight at lower rates (0.5 kg/day; red line and bar). ADG: Average daily gain; BW: body weight; LG: low- gain; HG: high-gain.

To identify the developmental window in which heifers are most responsive to the nutritional programming of puberty, we employed a stair-step nutritional regimen involving alternate periods of dietary energy-restriction and re-feeding during juvenile development. Interestingly, we observed that heifers that gained body weight at high rates between 4 and 6.5 months of age, and were subsequently subjected to a marked restriction in feed intake between 6.5 and 9 months of age, still attained early puberty (<12 months of age) at rates comparable to heifers fed a high- concentrate diet continuously (Cardoso *et al*., 2014b). Similarly, *Bos taurus* heifers that were fed to gain body weight at a relatively high rate between 126 and 196 days of age exhibited a high incidence of precocious puberty (Gasser *et al*., 2006a). However, puberty was not advanced to the same extent when heifers were fed a similar diet later during juvenile development. Collectively, these results demonstrate that during early development, plausibly between 4 and 9 months of age, heifers are more sensitive to the pubertal acceleration effects of nutrition (Fig. 1).

### Metabolic signals and neuroendocrine maturation

Nutritional regimens that promote a relative high rate of body weight gain (1 kg/day) are accompanied by greater adiposity and increased circulating concentrations of the metabolic hormones leptin, insulin, and insulin-like growth factor 1 (IGF1) when compared to regimens that promote growth at a slower rate (0.5 kg/day; Allen *et al*., 2012; Cardoso *et al*., 2014b; Alves *et al*., 2015). We hypothesized that this positive metabolic profile induced by increased body weight gain would promote modifications in the reproductive neuroendocrine system, ultimately resulting in increased pulsatile release of GnRH and LH, which are required for first ovulation. Using the same research model of accelerated growth during the juvenile period, we characterized the secretion of GnRH and LH in prepubertal heifers by measuring the concentrations of GnRH in third-ventricle cerebrospinal fluid (IIIV-CSF) and LH in the peripheral blood, respectively (Cardoso *et al*., 2014a). As expected, we observed that all pulses of LH in blood plasma were preceded by a GnRH pulse in the IIIV-CSF and, more importantly, pulse frequency of GnRH and LH were greater in heifers gaining 1 kg/day when compared to heifers gaining 0.5 kg/day between 4 and 8 months of age. These observations further indicate that increased nutrition during the juvenile period advances puberty by promoting the maturation of the reproductive neuroendocrine axis, thus hastening the pubertal increase in the pulsatile release of GnRH/LH.

## Postnatal programming of hypothalamic signalling pathways

A growing body of evidence indicates that metabolic factors associated with the individual’s nutritional status can influence the activity of GnRH neurons. Among metabolic factors, leptin plays a critical role in conveying nutritional information to the neuroendocrine axis and controlling pubertal progression in heifers (Zieba *et al*., 2005). However, the long form of the leptin receptor (ObRb), the main receptor isoform involved in activation of intracellular signaling, is not present on GnRH neurons (Quennell *et al*., 2009). Therefore, it has been postulated that leptin signaling influences GnRH neuronal activity via an upstream neuronal network that ultimately targets GnRH neurons (Barb and Kraeling, 2004; Ratra and Elias, 2014). Two different neuronal populations located in the ARC that contain ObRb (Cheung *et al*., 1997; Elmquist *et al*., 1998) and directly regulate the function of GnRH neurons (Roa and Herbison, 2012) have been established as main components of this network: the neuropeptide Y/agouti-related peptide (NPY/AgRP) and the proopiomelanocortin/cocaine- and amphetamine- regulated transcript (POMC/CART) neurons. In addition, because the ARC population of kisspeptin neurons is involved in the control of GnRH pulsatile release (Li *et al*., 2009) and kisspeptin synthesis is responsive to metabolic cues (Castellano *et al*., 2011), kisspeptin neurons in the ARC are also likely to be involved in the nutritional regulation of puberty.

### Neuropeptide Y/AgRP pathway

**Figure 2:**
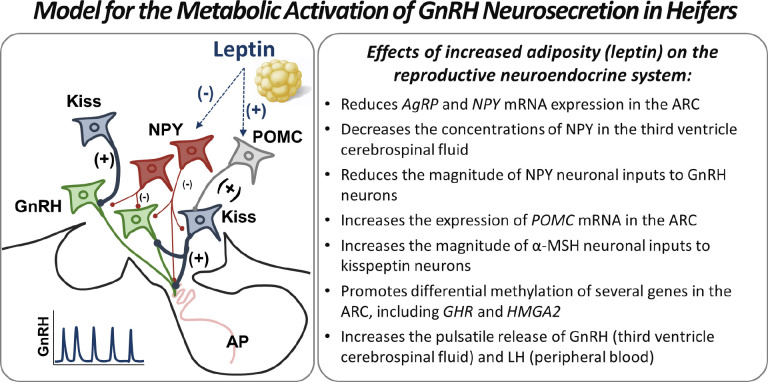
Neuroendocrine model for the metabolic activation of GnRH secretion during pubertal maturation in heifers. Left Panel: Representative scheme of neuronal pathways in the arcuate nucleus (ARC) that mediate the effects of leptin on GnRH pulsatile secretion. The adipocyte-derived hormone leptin inhibits (-) neuropeptide Y (NPY) and stimulates (+) proopiomelanocortin (POMC) neurons in the ARC. Consequently, the inhibitory (-) effects of NPY on GnRH neurons are diminished (thickness of lines relates to the intensity of the stimulus). POMC neurons project to kisspeptin (Kiss) neurons in the ARC, which stimulate (+) the pulsatile release of GnRH. The suppression of inhibitory (NPY) and increase in stimulatory (POMC and Kiss) pathways promote an increase in the frequency of GnRH pulses. GnRH neurons project to the hypophyseal portal circulation, allowing the access of this neurohormone to the anterior pituitary (AP), where it stimulates the secretion of gonadotropins. **Right Panel:** Summary of the effects of increased rates of body weight gain during the juvenile period on the reproductive neuroendocrine system in heifers.

The NPY/AgRP neuronal population secretes two main orexigenic neuropeptides: neuropeptide Y (NPY) and agouti-related peptide (AgRP), both secreted predominantly in conditions of low energy balance (McShane *et al*., 1992; Hahn *et al*., 1998). Experiments in mature cows have demonstrated that NPY has an inhibitory effect on GnRH release (Gazal *et al*., 1998) and, although not tested in cattle, AgRP has been shown to suppress GnRH release in sheep (Miller *et al*., 2007) and monkeys (Vulliémoz *et al*., 2005). Both neuropeptides, NPY and AgRP, are important inhibitory signals to GnRH secretion during prepubertal development and play a key role in controlling the timing of pubertal maturation in females (Pierroz *et al*., 1995; Egan *et al*., 2017). Our studies in prepubertal heifers have shown that increased body weight gain between 4 and 8 months of age (juvenile period) reduces *AgRP* (Allen *et al*., 2012) and *NPY* (Allen *et al*., 2012; Alves *et al*., 2015) mRNA expression in the ARC, decreases the concentrations of NPY in the cerebrospinal fluid collected from the third ventricle of the brain (Cardoso *et al*., 2014a), and reduces the magnitude of NPY neuronal inputs to GnRH neurons (Alves *et al*., 2015). Altogether, our findings in the bovine female support the notion that NPY and AgRP inhibit GnRH pulsatile release during the prepubertal period and nutritional regimens that promote accelerated rates of body weight gain during juvenile development can attenuate the NPY/AgRP inhibitory tone, thus facilitating pubertal maturation (Fig. 2).

### Proopiomelanocortin pathway

The POMC gene expressed in POMC/CART neurons encodes several peptides, including the anorexigenic alpha-melanocyte stimulating hormone (α- MSH), which is produced primarily during periods of positive energy balance (Cone, 1999). In rodents, α- MSH was shown to elicit a direct stimulatory effect on GnRH neurons (Leranth *et al*., 1988; Roa and Herbison, 2012) and administration of a melanocortin agonist stimulated LH secretion in the female sheep (Backholer *et al*., 2009). Notably, the melanocortin 4 receptor (MC4R) is antagonized by AgRP (Ollmann *et al*., 1997), indicating that AgRP may inhibit GnRH secretion not only directly but also indirectly via the melanocortin system. In our studies, the expression of *POMC* mRNA and α-MSH immunostaining in the ARC were both increased in heifers that gained body weight at accelerated rates during juvenile development (Allen *et al*., 2012; Cardoso *et al*., 2015). However, the number of α-MSH-immunopositive contacts on GnRH neurons was moderately low and was not affected by the nutritional status during early development (Cardoso *et al*., 2015). Collectively, these results suggest that the nutritional acceleration of puberty in heifers may require increased signaling of α-MSH in the hypothalamus, but not necessarily involving an increase in direct α-MSH stimulation of GnRH neurons.

### Kisspeptin pathway

There is emerging evidence indicating that the ARC population of kisspeptin neurons is involved in the nutritional control of reproductive function in females (Castellano *et al*., 2011). While a subset of kisspeptin neurons in the ARC contain ObRb (Backholer *et al*., 2010), leptin induction of STAT3 phosphorylation, a major intracellular signaling mechanism induced by leptin, is absent in kisspeptin neurons in sheep (Louis *et al*., 2011). Thus, the effects of leptin on kisspeptin expression and neuronal activity appear to be mediated indirectly via an upstream network of neurons (Donato *et al*., 2011; Manfredi-Lozano *et al*., 2016). In our studies in heifers, we tested if the number of close contacts of NPY- or α-MSH-containing fibers on kisspeptin neurons in the ARC would be associated with the nutritional regulation of puberty in heifers. While the intensity of NPY axonal contacts on kisspeptin neurons was not affected by nutritional status (Alves *et al*., 2015), accelerated rates of body weight gain resulted in an increased number of α-MSH immunopositive contacts on kisspeptin neurons and a greater percentage of kisspeptin neurons innervated by α-MSH fibers (Cardoso *et al*., 2015). Prepubertal heifers subjected to accelerated rates of body weight gain also exhibited reduced *Kiss1* mRNA content in the ARC (Alves *et al*., 2015; Cardoso *et al*., 2015), suggesting a possible influence of α-MSH on *Kiss1* gene expression during the juvenile period. Notably, a MTII melanocortin agonist has been shown to reduce *Kiss1* mRNA in the ARC of sheep (Backholer *et al*., 2009). In intact female rats, the developmental pattern of *Kiss1* expression in the ARC follows a U-like pattern, with expression declining from intermediate (infantile period), to minimal (juvenile period), followed by a postpubertal increase to maximum expression during adulthood (Cao and Patisaul, 2011). Therefore, reduced *Kiss1* expression in the ARC may indicate a more advanced stage of development of this neuronal population in heifers fed a higher plane of nutrition. Additionally, changes in *Kiss1* mRNA abundance in the ARC may result from the negative effects of estradiol on *Kiss1* expression (Smith, 2009), since steroidogenic capacity and circulating levels of estradiol increase as heifers approach puberty (Gasser *et al*., 2006b). Despite the observed changes in *Kiss1* mRNA expression, the number of kisspeptin-immunopositive neurons in the ARC was not affected by nutritional status in our studies (Alves *et al*., 2015; Cardoso *et al*., 2015). Based on those findings, the role of ARC kisspeptin neurons in the nutritional programming of puberty in heifers is still not fully understood. However, interactions between POMC and kisspeptin neurons appear to be relevant during this process (Fig. 2).

### Other pathways

In addition to changes in neuronal populations and neuropeptide signaling as discussed above, the ARC encompasses other cell types and molecules impacting a variety of cellular processes that regulate GnRH secretion, including receptors and transcription factors. In two of our studies in prepubertal heifers, we obtained a comprehensive survey of the different cellular populations of the ARC by isolating ARC tissue from within hypothalamic sections. In one experiment (Allen *et al*., 2012), using microarray analyses to assess mRNA abundance, we observed that different nutritional regimens applied during the juvenile period promoted differential expression of a large number of genes, including those encoding for prolactin-releasing hormone receptor (*PRLHR*) and growth hormone receptor (*GHR*). In addition, we observed differential expression of genes associated with control of feed intake and metabolism (*NPY, AgRP*, and *POMC*), as well as genes involved in synaptic vesicle transport, axonal growth and neuronal plasticity. These findings, in conjunction with observed changes in neuronal projections (Alves *et al*., 2015; Cardoso *et al*., 2015), support the premise that neuronal remodeling plays a significant role in programming the timing of puberty in heifers. The well-established neurotropic actions of leptin are likely to be involved in this process (Bouret *et al*., 2004).

In a follow-up study (Alves *et al*., 2016), we investigated if DNA methylation (key epigenetic mechanism for regulation of gene expression) in the ARC was influenced by nutritional status during the juvenile period. Differential methylation was found in several genes, including *GHR* and *HMGA2* (a gene that encodes a chromatin-associated protein that modulates transcription), which were hypermethylated in heifers that gained body weight at higher rates. Importantly, the hypermethylation of these genes was associated with a reduced abundance of their mRNA expression levels. Growth hormone has been previously reported to play a role in pubertal development in heifers (Simpson *et al*., 1991) and its action in the ARC appears to be linked to the control of NPY synthesis (Chan *et al*., 1996). Moreover, a genome-wide association study (GWAS) has indicated that *HMGA2* is one prominent gene involved in the process of pubertal maturation in heifers (Fortes *et al*., 2011), therefore, further investigation of its role in the nutritional programming of puberty is warranted. While the exact mechanisms remain unknown, this initial investigation of DNA methylation supports the premise that epigenetic alterations promoted by increased body weight gain during the juvenile period may represent a suitable mechanism by which nutrition can change functionality of the ARC cellular machinery, thus facilitating the activation of the reproductive neuroendocrine axis.

## Prenatal programming of hypothalamic signalling pathways

During the past decade, the developmental origins of health and disease (DOHaD) hypothesis by Barker and colleagues has attracted considerable attention to the concept of fetal programming (Barker, 2007). The DOHaD hypothesis gained momentum particularly after the emergence of epidemiological data from the 1944- 1945 Dutch famine cohort demonstrating that maternal malnutrition during gestation is associated with a marked increase in the risks of the offspring for developing cardiovascular and metabolic diseases (Ravelli *et al*., 1976). These findings, in conjunction with subsequent clinical and animal studies (Barker, 2004; Gluckman and Hanson, 2004; Nijland *et al*., 2008), demonstrate that the perinatal period, a period in which organogenesis and tissue differentiation occur through a tightly controlled and timed process, is a critical window of opportunity for programming the offspring’s phenotype. In recent years, it has become evident that the mechanisms underlying the developmental origins of the adult phenotype require reprogramming of the epigenome by environmental factors (Ganu *et al*., 2012; Trevino *et al*., 2015). This is possible during fetal life due to the plasticity that allows the developing organism to adopt a phenotype that best suits the environment. From a neuroendocrine standpoint, studies in laboratory rodents have shown that maternal nutrition during gestation modulates the hypothalamic neurocircuitries controlling GnRH pulsatile release, thus programing pubertal maturation in the female offspring (Léonhardt *et al*., 2003; Iwasa *et al*., 2010; Sanchez-Garrido *et al*., 2013). In cattle, however, the concept of prenatal programing of the reproductive neuroendocrine axis remains virtually unexplored. For the past several years, our research group has developed a bovine model to study the interactive effects of prenatal and early postnatal nutrition on reproductive function in the female offspring. To accomplish this, *Bos indicus*- influenced (Brahman × Hereford; Brangus) cows bearing female pregnancies were fed to achieve thin, moderate, or obese body condition (BC) by approximately 6 months of gestation (second trimester) and maintained at the target BC until calving. Heifer offspring from each maternal group were then weaned at approximately 3.5 months of age and assigned randomly to be fed to achieve a relatively low (0.5 kg/day) or high (1 kg/day) rate of body weight gain until 8 months of age. While results of most of our studies using this animal model remain pending, initial findings are summarized in the following sections.

### Leptin transport across the blood-brain barrier

As discussed previously, leptin is a critical metabolic hormone conveying nutritional information to the neuroendocrine axis and controlling pubertal development in females. Previous studies in sheep have shown that changes in nutritional status can modulate the transport of leptin across the blood-brain barrier. For example, in a study in which obese and lean adult sheep were fed to either gain or lose body weight, it was observed that obese animals had impaired transport of leptin across the blood-brain barrier (Adam and Findlay, 2010). Moreover, the transport of peripherally- administered leptin across the blood-brain barrier in obese sheep was not reversed following significant body weight loss. These and other data suggest that exposure of animals to a hyperleptinemic environment, such as that expected to occur in the fetus of a dam on a high nutritional plane, can result in irreversible physiological changes within the blood-brain barrier. Thus, we hypothesized that heifer offspring of dams with broadly varying degrees of BC may develop a leptin resistant state due to structural alterations in the blood-brain barrier.

To test this hypothesis, we investigated the expression of different isoforms of the leptin receptor in the choroid plexus of heifers subjected to the different maternal (thin, moderate, and obese) and postnatal (low and high) nutritional treatments. Importantly, the transport of leptin across the blood-brain barrier has been shown to depend on expression of the short form of the leptin receptor, which acts as a leptin transporter in endothelial cells (Banks, 2001). We found that prenatal nutrient restriction significantly reduced the mRNA abundance of the short form of the leptin receptor ObRc in the choroid plexus of heifers at 8 months of age (Zhang *et al*., 2017). Moreover, the expression of total leptin receptor (ObRt) was also reduced in the choroid plexus of heifers subjected to prenatal undernutrition. Interestingly, postnatal nutrient restriction increased the expression of ObRb, the long form of the leptin receptor, in the choroid plexus of heifers subjected to prenatal undernutrition but not in heifers subjected to other prenatal nutritional treatments (moderate or obese). Collectively, these results indicate that undernutrition during pregnancy interacts with postnatal nutrition to modulate the expression of the different isoforms of the leptin receptor in the choroid plexus of prepubertal heifers. The significance of changes in leptin receptor expression in regard to leptin transport across the blood- brain barrier is currently being investigated.

### Neuropeptide y pathway

Using hypothalamic tissue from the same group of heifers discussed above, we investigated the interactive effects of prenatal and postnatal nutrition on the extent of NPY (inhibitory) projections toward GnRH neurons. While none of the treatment combinations altered the number of GnRH neurons, reduced rates of body weight gain during postnatal development increased the proportion of GnRH neurons in close apposition to NPY-containing fibers (Zhang *et al*., 2017). Notably, these effects were significantly greater in heifers from nutritionally-restricted dams, suggesting that prenatal undernutrition may amplify the effects of postnatal nutrition modulating the extent of NPY neuronal projections to GnRH neurons. While the functional relevance of this phenomenon remains to be determined, it is likely that the effects of prenatal undernutrition on NPY projections to GnRH neurons will hinder the process of pubertal maturation in heifers.

## Ongoing and future studies

In our ongoing and future studies, we will further examine the interactive effects of prenatal nutrition with nutritional treatments imposed postnatally during the early juvenile period on hypothalamic processes controlling puberty. These include changes in expression of key genes (*e.g., Kiss1, POMC*, and *NPY*) in specific hypothalamic nuclei, epigenetic modification (*e.g.,* DNA methylation patterns), morphological development of neuronal pathways that modulate activity of the GnRH pulse generator, leptin signaling in the hypothalamus, among others. Importantly, we currently have available a large group of nutritionally- programmed heifers that will allow us to characterize the interactive effects of prenatal and early postnatal nutrition on multiple physiological processes, such as leptin transport across the blood-brain barrier, central release of neuropeptides, pulsatile secretion of GnRH and LH, responsiveness to estradiol negative and positive feedback, and estrous cycle events associated with postpubertal maturation. Moreover, because organizational changes brought about through perinatal nutritional programming are believed to be imprinted in the genome, they are likely to be associated with consistent physiological events that may, in some cases, be manifested only later in life. Thus, these nutritionally-programmed females represent a valuable resource for evaluating the long-term consequences of perinatal nutrition on reproductive function of sexually mature animals. We believe that the perinatal nutritional environment can program how the reproductive neuroendocrine axis of sexually mature heifers responds to leptin and other metabolic/hormonal factors, particularly under unfavorable metabolic conditions.

Finally, it will be important in the future to determine the transgenerational effects of perinatal nutrition in the bovine female. Studies in rodents have shown that experimental manipulation of the nutritional plane during the perinatal period can significantly impact the reproductive and metabolic phenotypes of subsequent generations (Pinheiro *et al*., 2008; Pentinat *et al*., 2010; Burdge *et al*., 2011). While these studies are difficult to perform in cattle due to the long generation interval, a better understanding of this process can have important implications for lifetime animal health and productivity.
